# Tetra­kis(2-amino-6-methyl­pyridinium) hexa­chloridobismuthate(III) chloride monohydrate

**DOI:** 10.1107/S1600536809025446

**Published:** 2009-07-08

**Authors:** Zhen Yang, Gang Chen, Wei Xu, Zheng Fan

**Affiliations:** a College of Pharmaceutical Sciences, Zhejiang University of Technology, Hangzhou 310014, People’s Republic of China; b College of Biological & Environmental Engineering, Zhejiang University of Technology, Hangzhou 310014, People’s Republic of China

## Abstract

The asymmetric unit of the title compound, (C_6_H_9_N_2_)_4_[BiCl_6_]Cl·H_2_O, contains four protonated 2-amino-6-methyl­pyridine (HAMP) cations and two-halves of two [BiCl_6_]^3−^ anions, together with one water mol­ecule and one chloride anion. The Bi^III^ atoms are hexa­coordinated by Cl atoms, forming distorted octa­hedral geometries. In the crystal structure, intra­molecular O—H⋯Cl and N—H⋯Cl, and inter­molecular O—H⋯Cl and N—H⋯O inter­actions link the mol­ecules into a three-dimensional network.

## Related literature

For related structures, see: Albrecht *et al.* (2003 ▶); Feng *et al.* (2007 ▶); Inuzuka & Fujimoto (1986 ▶, 1990 ▶); Ishikawa *et al.* (2002 ▶); Jin *et al.* (2000 ▶, 2001 ▶, 2005 ▶); Luque *et al.* (1997 ▶); Nahringbauer & Kvick (1977 ▶); Ren *et al.* (2002 ▶); Rivas *et al.* (2003 ▶); Salwa *et al.* (2008 ▶); Xu *et al.* (2006 ▶).
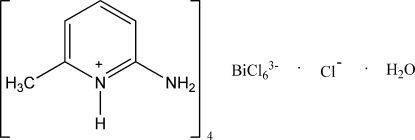


## Experimental

### 

#### Crystal data

(C_6_H_9_N_2_)_4_[BiCl_6_]Cl·H_2_O
*M* *_r_* = 911.75
Triclinic, 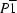

*a* = 10.3345 (7) Å
*b* = 10.7605 (7) Å
*c* = 17.2673 (11) Å
α = 100.3370 (10)°
β = 103.7370 (10)°
γ = 99.2280 (10)°
*V* = 1793.1 (2) Å^3^
*Z* = 2
Mo *K*α radiation
μ = 5.47 mm^−1^
*T* = 273 K
0.42 × 0.31 × 0.25 mm

#### Data collection

Bruker SMART APEX area-detector diffractometer
Absorption correction: multi-scan (*SADABS*; Bruker, 2000 ▶) *T*_min_ = 0.153, *T*_max_ = 0.185 (expected range = 0.211–0.255)
9489 measured reflections
6240 independent reflections
5171 reflections with *I* > 2σ(*I*)
*R*_int_ = 0.016

#### Refinement

*R*[*F* ^2^ > 2σ(*F* ^2^)] = 0.026
*wR*(*F* ^2^) = 0.070
*S* = 1.07
6240 reflections
373 parameters
3 restraints
H-atom parameters constrained
Δρ_max_ = 0.55 e Å^−3^
Δρ_min_ = −1.13 e Å^−3^

### 

Data collection: *SMART* (Bruker, 2000 ▶); cell refinement: *SAINT* (Bruker, 2000 ▶); data reduction: *SAINT*; program(s) used to solve structure: *SHELXTL* (Sheldrick, 2008 ▶); program(s) used to refine structure: *SHELXL97* (Sheldrick, 2008 ▶); molecular graphics: *SHELXTL*; software used to prepare material for publication: *SHELXTL*.

## Supplementary Material

Crystal structure: contains datablocks I, global. DOI: 10.1107/S1600536809025446/hk2720sup1.cif

Structure factors: contains datablocks I. DOI: 10.1107/S1600536809025446/hk2720Isup2.hkl

Additional supplementary materials:  crystallographic information; 3D view; checkCIF report

## Figures and Tables

**Table 1:** Hydrogen-bond geometry (Å, °)

*D*—H⋯*A*	*D*—H	H⋯*A*	*D*⋯*A*	*D*—H⋯*A*
O1—H1*WB*⋯Cl7	0.825	2.28	3.051 (3)	157
N2—H2*B*⋯Cl5	0.86	2.65	3.432 (3)	151
N4—H4*B*⋯Cl2	0.86	2.48	3.307 (3)	163
N5—H5⋯Cl7	0.86	2.21	3.059 (3)	168
N7—H7⋯Cl4	0.86	2.38	3.204 (3)	161
N8—H8*B*⋯Cl1	0.86	2.51	3.343 (3)	164
O1—H1*WA*⋯Cl3^i^	0.828	2.49	3.290 (3)	163
N1—H1⋯O1^ii^	0.86	1.91	2.774 (3)	177

Symmetry codes: (i) 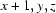; (ii) 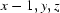.

**Fig. 1.**
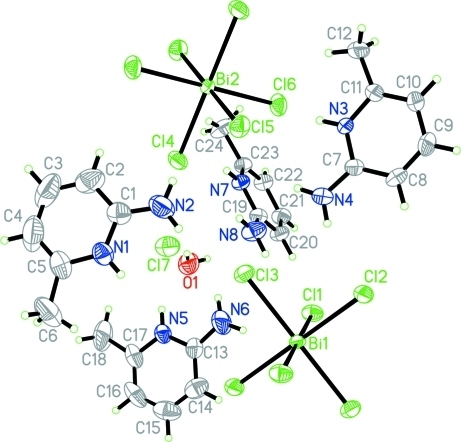
The molecular structure of the title molecule, with the atom-numbering scheme. Displacement ellipsoids are drawn at the 40% probability level.

**Fig. 2.**
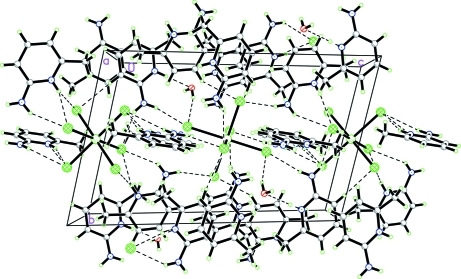
A packing diagram viewed down along the a axis. Hydrogen bonds are shown as dashed lines.

## supplementary crystallographic information

### Comment

During the past decade, a series of 2-amino-substituted pyridine compounds have been investigated in which the 2-aminopyridines act as ligands or protonated cations (Ren *et al.*, 2002; Rivas *et al.*, 2003; Luque *et al.*, 1997; Albrecht *et al.*, 2003). Among them, the tautomerism phenomenon of 2-aminopyridine derivatives has been proved by *x*-ray diffraction, such as 2-amino-6-methylpyridinium chloride (Jin *et al.*, 2000) and 2-amino-6-methylpyridinium neoabietate (Jin *et al.*, 2005). All the above studies provide important references to further research into 2-amino pyridines. We report herein the crystal structure of the title compound.

The asymmetric unit of the title compound, (Fig. 1), contains four protonated 2-amino-6-methyl-pyridine (HAMP) cations and two-halves of crystallographically independent [BiCl_6_]^3-^ anions, together with one water molecule and one chloride anion. The bismuth atoms are hexa-coordinated by chloride atoms, forming distorted-octahedral geometries. Intramolecular O-H···Cl and N-H···Cl interactions (Table 1) link the cations, anions and water molecule.

The average value of Bi—Cl bond distance [2.7061 Å] observed in the [BiCl_6_]^3-^ anion is shorter than the corresponding average values of [2.7130 Å] (Salwa *et al.*, 2008) and [2.7150 Å] (Xu *et al.*, 2006). In the cation, the N4—C11 bond [1.334 (5) Å] is shorter than the N3—C11 [1.341 (5) Å] and N3—C7 [1.358 (5) Å] bonds, and the C10—C11 [1.384 (6) Å] and C8—C9 [1.402 (6) Å] bonds are significantly longer than C9—C10 [1.362 (7) Å] and C7—C8 [1.342 (6) Å] bonds, in which they are similar to those in the HAMP cation (C_6_H_9_N_2_)_2_[Sb_2_Cl_6_O] (Feng *et al.*, 2007). In contrast, in the solid state structure of 2-amino-6-methyl-pyridine (AMP), the N—C bond out of the ring is clearly longer than that in the ring (Nahringbauer *et al.*, 1977). The geometric features of HAMP cation [N7/N8/C19/C24] resemble those observed in other 2-aminopyridine structures (Jin *et al.*, 2001) that are believed to be involved in amine-imine tautomerism (Inuzuka *et al.*, 1986; Inuzuka *et al.*, 1990; Ishikawa *et al.*, 2002). Similar features are also observed in other HAMP cations.

In the crystal structure (Fig. 2), intramolecular O-H···Cl and N-H···Cl and intermolecular O-H···Cl and N-H···O interactions (Table 1) link the molecules into a three-dimensional network.

### Experimental

For the preparation of the title compound, AMP, aqueous HCl and BiCl_3_ in a molar ratio of 4:4:1 were mixed and dissolved in water (20 ml). The mixture was stirred and heated until a clear solution was resulted. Crystals suitable for X-ray analysis were obtained by gradual evaporation of excess water over a period of one week at 300 K. Analysis: C 31.65; H 4.13; N 12.32. calc. for Bi_1_C_24_H_34_N_8_O_1_Cl_7_: C 31.61; H 4.17; N 12.29 IR Spectrum (KBr, cm^-1^): 3411(*s*), 3295 (*s*), 3195 (*m*), 3090 (*m*), 1656 (*versus*), 1630 (w), 1565 (w), 1474 (w), 1392 (*m*), 1309 (*m*), 1174 (w), 1042 (w), 997 (w), 793 (*m*), 715 (w), 612 (w), 564 (w), 421 (*m*).

### Refinement

H atoms were positioned geometrically with O-H = 0.8249 and 0.8278 Å (for H_2_O), N-H = 0.86 Å (for NH and NH_2_) and C-H = 0.93 and 0.96 Å for aromatic and methyl H atoms, respectively, and constrained to ride on their parent atoms, with U_iso_(H) = xU_eq_(C,N,O), where x = 1.5 for methyl H and x = 1.2 for all other H atoms.

### Crystal data

**Table:**

(C_6_H_9_N_2_)_4_[BiCl_6_]Cl·H_2_O	*Z* = 2
*M**_r_* = 911.75	*F*(000) = 896.0
Triclinic, *P*1	*D*_x_ = 1.689 Mg m^−^^3^
Hall symbol: -P 1	Mo *K*α radiation, λ = 0.71073 Å
*a* = 10.3345 (7) Å	Cell parameters from 3117 reflections
*b* = 10.7605 (7) Å	θ = 2.2–25.1°
*c* = 17.2673 (11) Å	µ = 5.47 mm^−^^1^
α = 100.337 (1)°	*T* = 273 K
β = 103.737 (1)°	Block, colorless
γ = 99.228 (1)°	0.42 × 0.31 × 0.25 mm
*V* = 1793.1 (2) Å^3^	

### Data collection

**Table:**

Bruker SMART APEX area-detector diffractometer	6240 independent reflections
Radiation source: fine-focus sealed tube	5171 reflections with *I* > 2σ(*I*)
graphite	*R*_int_ = 0.016
φ and ω scans	θ_max_ = 25.0°, θ_min_ = 1.3°
Absorption correction: multi-scan (*SADABS*; Bruker, 2000)	*h* = −10→12
*T*_min_ = 0.153, *T*_max_ = 0.185	*k* = −9→12
9489 measured reflections	*l* = −20→20

### Refinement

**Table:**

Refinement on *F*^2^	Primary atom site location: structure-invariant direct methods
Least-squares matrix: full	Secondary atom site location: difference Fourier map
*R*[*F*^2^ > 2σ(*F*^2^)] = 0.026	Hydrogen site location: inferred from neighbouring sites
*wR*(*F*^2^) = 0.070	H-atom parameters constrained
*S* = 1.07	*w* = 1/[σ^2^(*F*_o_^2^) + (0.0359*P*)^2^ + 0.5382*P*] where *P* = (*F*_o_^2^ + 2*F*_c_^2^)/3
6240 reflections	(Δ/σ)_max_ < 0.001
373 parameters	Δρ_max_ = 0.55 e Å^−^^3^
3 restraints	Δρ_min_ = −1.13 e Å^−^^3^

### Special details

**Table:**

Geometry. All e.s.d.'s (except the e.s.d. in the dihedral angle between two l.s. planes) are estimated using the full covariance matrix. The cell e.s.d.'s are taken into account individually in the estimation of e.s.d.'s in distances, angles and torsion angles; correlations between e.s.d.'s in cell parameters are only used when they are defined by crystal symmetry. An approximate (isotropic) treatment of cell e.s.d.'s is used for estimating e.s.d.'s involving l.s. planes.
Refinement. Refinement of *F*^2^ against ALL reflections. The weighted *R*-factor *wR* and goodness of fit *S* are based on *F*^2^, conventional *R*-factors *R* are based on *F*, with *F* set to zero for negative *F*^2^. The threshold expression of *F*^2^ > σ(*F*^2^) is used only for calculating *R*-factors(gt) *etc*. and is not relevant to the choice of reflections for refinement. *R*-factors based on *F*^2^ are statistically about twice as large as those based on *F*, and *R*- factors based on ALL data will be even larger.

### Fractional atomic coordinates and isotropic or equivalent isotropic displacement parameters (Å^2^)

**Table:**

	*x*	*y*	*z*	*U*_iso_*/*U*_eq_	
Bi1	0.5000	0.5000	0.5000	0.03208 (7)	
Bi2	0.5000	0.5000	0.0000	0.03407 (7)	
Cl1	0.76385 (11)	0.55587 (11)	0.49688 (6)	0.0503 (3)	
Cl2	0.46967 (13)	0.73844 (10)	0.48394 (7)	0.0537 (3)	
Cl3	0.42723 (11)	0.42048 (10)	0.33422 (6)	0.0489 (3)	
Cl4	0.56519 (12)	0.32481 (10)	0.08902 (7)	0.0573 (3)	
Cl5	0.33494 (12)	0.56458 (11)	0.09478 (7)	0.0547 (3)	
Cl6	0.70316 (12)	0.68084 (10)	0.10950 (7)	0.0599 (3)	
Cl7	0.88297 (16)	0.15531 (15)	0.23702 (9)	0.0826 (4)	
O1	1.1851 (4)	0.1659 (3)	0.3096 (2)	0.0857 (11)	
H1WB	1.1129	0.1812	0.2849	0.103*	
H1WA	1.2497	0.2292	0.3265	0.103*	
N1	0.1776 (4)	0.0607 (4)	0.1493 (3)	0.0592 (11)	
H1	0.1767	0.0927	0.1985	0.071*	
N2	0.2421 (5)	0.2693 (4)	0.1378 (3)	0.0810 (13)	
H2A	0.2425	0.2963	0.1878	0.097*	
H2B	0.2629	0.3238	0.1095	0.097*	
N3	0.5100 (4)	0.8575 (3)	0.20673 (19)	0.0474 (9)	
H3A	0.5145	0.7911	0.1722	0.057*	
N4	0.4851 (5)	0.7216 (4)	0.2933 (2)	0.0781 (14)	
H4A	0.4900	0.6588	0.2563	0.094*	
H4B	0.4747	0.7079	0.3392	0.094*	
N5	0.8447 (4)	0.0458 (4)	0.3837 (3)	0.0502 (9)	
H5	0.8607	0.0669	0.3407	0.060*	
N6	0.8249 (5)	0.2543 (4)	0.4248 (3)	0.0840 (14)	
H6D	0.8403	0.2684	0.3800	0.101*	
H6E	0.8112	0.3155	0.4593	0.101*	
N7	0.8615 (3)	0.4698 (3)	0.2053 (2)	0.0421 (8)	
H7	0.7861	0.4446	0.1672	0.050*	
N8	0.7323 (4)	0.4673 (4)	0.2964 (2)	0.0664 (11)	
H8A	0.6604	0.4426	0.2556	0.080*	
H8B	0.7251	0.4784	0.3456	0.080*	
C1	0.2094 (5)	0.1432 (6)	0.1040 (3)	0.0618 (13)	
C2	0.2063 (7)	0.0900 (8)	0.0237 (4)	0.094 (2)	
H2	0.2286	0.1435	−0.0100	0.112*	
C3	0.1711 (9)	−0.0385 (10)	−0.0048 (5)	0.124 (3)	
H3	0.1641	−0.0735	−0.0593	0.149*	
C4	0.1446 (9)	−0.1207 (8)	0.0466 (6)	0.130 (3)	
H4	0.1259	−0.2099	0.0274	0.156*	
C5	0.1466 (6)	−0.0699 (6)	0.1232 (4)	0.0808 (17)	
C6	0.1129 (8)	−0.1460 (6)	0.1820 (5)	0.126 (3)	
H6A	0.1219	−0.0885	0.2333	0.189*	
H6B	0.1741	−0.2038	0.1902	0.189*	
H6C	0.0209	−0.1951	0.1606	0.189*	
C7	0.4938 (5)	0.8408 (4)	0.2794 (2)	0.0474 (10)	
C8	0.4871 (5)	0.9476 (5)	0.3355 (3)	0.0513 (11)	
H8	0.4766	0.9398	0.3866	0.062*	
C9	0.4962 (5)	1.0642 (5)	0.3144 (3)	0.0547 (12)	
H9	0.4914	1.1366	0.3513	0.066*	
C10	0.5125 (5)	1.0763 (4)	0.2382 (3)	0.0510 (11)	
H10	0.5185	1.1565	0.2247	0.061*	
C11	0.5198 (5)	0.9730 (4)	0.1841 (3)	0.0452 (10)	
C12	0.5398 (6)	0.9723 (5)	0.1016 (3)	0.0676 (14)	
H12A	0.5410	0.8862	0.0753	0.101*	
H12B	0.6248	1.0290	0.1069	0.101*	
H12C	0.4665	1.0014	0.0692	0.101*	
C13	0.8223 (5)	0.1368 (5)	0.4407 (3)	0.0536 (12)	
C14	0.7972 (6)	0.1029 (6)	0.5099 (3)	0.0726 (16)	
H14	0.7820	0.1638	0.5504	0.087*	
C15	0.7949 (6)	−0.0189 (8)	0.5181 (4)	0.086 (2)	
H15	0.7779	−0.0417	0.5647	0.103*	
C16	0.8170 (6)	−0.1111 (6)	0.4591 (5)	0.084 (2)	
H16	0.8141	−0.1954	0.4655	0.101*	
C17	0.8431 (5)	−0.0771 (5)	0.3914 (4)	0.0673 (15)	
C18	0.8685 (7)	−0.1648 (6)	0.3227 (4)	0.110 (3)	
H18A	0.8842	−0.1179	0.2823	0.166*	
H18B	0.7906	−0.2347	0.2984	0.166*	
H18C	0.9471	−0.1988	0.3427	0.166*	
C19	0.8552 (4)	0.4883 (4)	0.2832 (2)	0.0434 (10)	
C20	0.9768 (5)	0.5280 (4)	0.3444 (3)	0.0513 (11)	
H20	0.9765	0.5424	0.3991	0.062*	
C21	1.0970 (5)	0.5458 (5)	0.3242 (3)	0.0513 (11)	
H21	1.1790	0.5707	0.3653	0.062*	
C22	1.0979 (5)	0.5268 (4)	0.2419 (3)	0.0482 (11)	
H22	1.1800	0.5408	0.2282	0.058*	
C23	0.9793 (4)	0.4882 (4)	0.1830 (3)	0.0428 (10)	
C24	0.9655 (5)	0.4629 (6)	0.0929 (3)	0.0738 (15)	
H24A	0.8707	0.4364	0.0633	0.111*	
H24B	1.0121	0.3957	0.0783	0.111*	
H24C	1.0048	0.5403	0.0793	0.111*	

### Atomic displacement parameters (Å^2^)

**Table:**

	*U*^11^	*U*^22^	*U*^33^	*U*^12^	*U*^13^	*U*^23^
Bi1	0.03694 (12)	0.03611 (12)	0.02651 (11)	0.00931 (9)	0.01195 (8)	0.00979 (8)
Bi2	0.03917 (13)	0.03067 (12)	0.02879 (11)	0.00442 (9)	0.00502 (9)	0.00606 (8)
Cl1	0.0429 (6)	0.0620 (7)	0.0507 (6)	0.0078 (5)	0.0165 (5)	0.0224 (5)
Cl2	0.0791 (8)	0.0442 (6)	0.0486 (6)	0.0211 (5)	0.0280 (6)	0.0170 (5)
Cl3	0.0524 (6)	0.0586 (7)	0.0340 (5)	0.0077 (5)	0.0125 (5)	0.0090 (4)
Cl4	0.0568 (7)	0.0446 (6)	0.0610 (7)	0.0003 (5)	−0.0039 (5)	0.0237 (5)
Cl5	0.0553 (7)	0.0571 (7)	0.0534 (6)	0.0082 (5)	0.0265 (5)	0.0047 (5)
Cl6	0.0538 (7)	0.0436 (6)	0.0631 (7)	0.0017 (5)	−0.0073 (5)	0.0026 (5)
Cl7	0.0804 (10)	0.0984 (11)	0.0720 (9)	0.0159 (8)	0.0196 (8)	0.0314 (8)
O1	0.075 (3)	0.089 (3)	0.078 (2)	−0.007 (2)	0.016 (2)	0.009 (2)
N1	0.054 (2)	0.054 (3)	0.070 (3)	0.0043 (19)	0.021 (2)	0.014 (2)
N2	0.101 (4)	0.066 (3)	0.091 (3)	0.015 (3)	0.046 (3)	0.033 (3)
N3	0.077 (3)	0.0354 (19)	0.0299 (17)	0.0145 (18)	0.0157 (17)	0.0055 (14)
N4	0.133 (4)	0.054 (3)	0.047 (2)	0.009 (3)	0.024 (2)	0.022 (2)
N5	0.050 (2)	0.041 (2)	0.058 (2)	0.0123 (17)	0.0093 (19)	0.0108 (18)
N6	0.113 (4)	0.050 (3)	0.098 (4)	0.029 (3)	0.040 (3)	0.015 (2)
N7	0.0369 (19)	0.053 (2)	0.0365 (18)	0.0087 (16)	0.0089 (15)	0.0119 (16)
N8	0.052 (2)	0.096 (3)	0.058 (2)	0.011 (2)	0.027 (2)	0.021 (2)
C1	0.047 (3)	0.073 (4)	0.071 (3)	0.008 (2)	0.025 (3)	0.021 (3)
C2	0.082 (5)	0.130 (7)	0.071 (4)	0.004 (4)	0.037 (4)	0.023 (4)
C3	0.112 (6)	0.144 (8)	0.083 (5)	−0.019 (6)	0.043 (5)	−0.037 (5)
C4	0.146 (8)	0.089 (6)	0.125 (7)	−0.025 (5)	0.059 (6)	−0.035 (5)
C5	0.072 (4)	0.059 (4)	0.101 (5)	−0.003 (3)	0.021 (3)	0.013 (3)
C6	0.143 (7)	0.078 (5)	0.148 (7)	−0.016 (4)	0.036 (6)	0.046 (5)
C7	0.056 (3)	0.047 (3)	0.039 (2)	0.006 (2)	0.013 (2)	0.0141 (19)
C8	0.056 (3)	0.067 (3)	0.031 (2)	0.013 (2)	0.015 (2)	0.007 (2)
C9	0.057 (3)	0.051 (3)	0.052 (3)	0.020 (2)	0.015 (2)	−0.004 (2)
C10	0.065 (3)	0.040 (3)	0.048 (3)	0.015 (2)	0.013 (2)	0.008 (2)
C11	0.052 (3)	0.041 (2)	0.045 (2)	0.012 (2)	0.014 (2)	0.014 (2)
C12	0.106 (4)	0.063 (3)	0.047 (3)	0.025 (3)	0.035 (3)	0.023 (2)
C13	0.048 (3)	0.048 (3)	0.059 (3)	0.011 (2)	0.008 (2)	0.006 (2)
C14	0.058 (3)	0.099 (5)	0.059 (3)	0.018 (3)	0.014 (3)	0.015 (3)
C15	0.060 (4)	0.117 (6)	0.086 (5)	0.010 (4)	0.013 (3)	0.056 (4)
C16	0.062 (4)	0.055 (4)	0.132 (6)	0.007 (3)	0.006 (4)	0.047 (4)
C17	0.055 (3)	0.043 (3)	0.097 (4)	0.008 (2)	0.009 (3)	0.016 (3)
C18	0.131 (6)	0.067 (4)	0.119 (5)	0.042 (4)	0.024 (5)	−0.020 (4)
C19	0.046 (2)	0.046 (2)	0.044 (2)	0.0137 (19)	0.017 (2)	0.0166 (19)
C20	0.056 (3)	0.062 (3)	0.035 (2)	0.012 (2)	0.008 (2)	0.015 (2)
C21	0.042 (3)	0.062 (3)	0.047 (3)	0.011 (2)	0.003 (2)	0.019 (2)
C22	0.039 (2)	0.062 (3)	0.047 (3)	0.014 (2)	0.014 (2)	0.016 (2)
C23	0.041 (2)	0.051 (3)	0.042 (2)	0.0134 (19)	0.0153 (19)	0.0137 (19)
C24	0.064 (3)	0.112 (5)	0.040 (3)	0.009 (3)	0.017 (2)	0.009 (3)

### Geometric parameters (Å, °)

**Table:**

Bi1—Cl1^i^	2.7121 (11)	C3—C4	1.401 (12)
Bi1—Cl1	2.7121 (11)	C3—H3	0.9300
Bi1—Cl2	2.6888 (10)	C4—C5	1.331 (10)
Bi1—Cl2^i^	2.6888 (10)	C4—H4	0.9300
Bi1—Cl3^i^	2.7175 (10)	C5—C6	1.484 (9)
Bi1—Cl3	2.7175 (10)	C6—H6A	0.9600
Bi2—Cl4	2.7066 (10)	C6—H6B	0.9600
Bi2—Cl5	2.7146 (10)	C6—H6C	0.9600
Bi2—Cl5^ii^	2.7146 (10)	C7—C8	1.386 (6)
Bi2—Cl6^ii^	2.6932 (10)	C8—C9	1.364 (7)
Bi2—Cl6	2.6932 (11)	C8—H8	0.9300
Bi2—Cl4^ii^	2.7066 (10)	C9—C10	1.390 (7)
O1—H1WA	0.8278	C9—H9	0.9300
O1—H1WB	0.8249	C10—C11	1.342 (6)
N1—C1	1.336 (6)	C10—H10	0.9300
N1—C5	1.357 (7)	C11—C12	1.487 (6)
N1—H1	0.8600	C12—H12A	0.9600
N2—C1	1.331 (6)	C12—H12B	0.9600
N2—H2A	0.8600	C12—H12C	0.9600
N2—H2B	0.8600	C13—C14	1.379 (8)
N3—C7	1.344 (5)	C14—C15	1.340 (9)
N3—C11	1.363 (5)	C14—H14	0.9300
N3—H3A	0.8600	C15—C16	1.376 (10)
N4—C7	1.340 (5)	C15—H15	0.9300
N4—H4A	0.8600	C16—C17	1.358 (9)
N4—H4B	0.8600	C16—H16	0.9300
N5—C13	1.348 (6)	C17—C18	1.481 (8)
N5—C17	1.350 (6)	C18—H18A	0.9600
N5—H5	0.8600	C18—H18B	0.9600
N6—C13	1.338 (6)	C18—H18C	0.9600
N6—H6D	0.8600	C19—C20	1.384 (6)
N6—H6E	0.8600	C20—C21	1.362 (7)
N7—C19	1.341 (5)	C20—H20	0.9300
N7—C23	1.358 (5)	C21—C22	1.402 (6)
N7—H7	0.8600	C21—H21	0.9300
N8—C19	1.334 (5)	C22—C23	1.342 (6)
N8—H8A	0.8600	C22—H22	0.9300
N8—H8B	0.8600	C23—C24	1.498 (6)
C1—C2	1.392 (8)	C24—H24A	0.9600
C2—C3	1.340 (11)	C24—H24B	0.9600
C2—H2	0.9300	C24—H24C	0.9600
			
Cl2—Bi1—Cl2^i^	180.0	C5—C6—H6A	109.5
Cl2—Bi1—Cl1^i^	88.75 (3)	C5—C6—H6B	109.5
Cl2^i^—Bi1—Cl1^i^	91.25 (3)	H6A—C6—H6B	109.5
Cl2—Bi1—Cl1	91.25 (3)	C5—C6—H6C	109.5
Cl2^i^—Bi1—Cl1	88.75 (3)	H6A—C6—H6C	109.5
Cl1^i^—Bi1—Cl1	180.0	H6B—C6—H6C	109.5
Cl2—Bi1—Cl3^i^	90.89 (3)	N4—C7—N3	117.9 (4)
Cl2^i^—Bi1—Cl3^i^	89.11 (3)	N4—C7—C8	123.9 (4)
Cl1^i^—Bi1—Cl3^i^	88.60 (3)	N3—C7—C8	118.2 (4)
Cl1—Bi1—Cl3^i^	91.40 (3)	C9—C8—C7	118.7 (4)
Cl2—Bi1—Cl3	89.11 (3)	C9—C8—H8	120.6
Cl2^i^—Bi1—Cl3	90.89 (3)	C7—C8—H8	120.6
Cl1^i^—Bi1—Cl3	91.40 (3)	C8—C9—C10	120.8 (4)
Cl1—Bi1—Cl3	88.60 (3)	C8—C9—H9	119.6
Cl3^i^—Bi1—Cl3	180.0	C10—C9—H9	119.6
Cl6^ii^—Bi2—Cl6	180.00 (5)	C11—C10—C9	120.5 (4)
Cl6^ii^—Bi2—Cl4^ii^	89.13 (3)	C11—C10—H10	119.8
Cl6—Bi2—Cl4^ii^	90.87 (3)	C9—C10—H10	119.8
Cl6^ii^—Bi2—Cl4	90.87 (3)	C10—C11—N3	117.5 (4)
Cl6—Bi2—Cl4	89.13 (3)	C10—C11—C12	126.2 (4)
Cl4^ii^—Bi2—Cl4	180.00 (3)	N3—C11—C12	116.3 (4)
Cl6^ii^—Bi2—Cl5	92.40 (4)	C11—C12—H12A	109.5
Cl6—Bi2—Cl5	87.60 (4)	C11—C12—H12B	109.5
Cl4^ii^—Bi2—Cl5	91.47 (4)	H12A—C12—H12B	109.5
Cl4—Bi2—Cl5	88.53 (4)	C11—C12—H12C	109.5
Cl6^ii^—Bi2—Cl5^ii^	87.60 (4)	H12A—C12—H12C	109.5
Cl6—Bi2—Cl5^ii^	92.40 (4)	H12B—C12—H12C	109.5
Cl4^ii^—Bi2—Cl5^ii^	88.53 (4)	N6—C13—N5	116.6 (5)
Cl4—Bi2—Cl5^ii^	91.47 (4)	N6—C13—C14	125.0 (5)
Cl5—Bi2—Cl5^ii^	180.00 (3)	N5—C13—C14	118.5 (5)
H1WB—O1—H1WA	114.5	C15—C14—C13	119.2 (6)
C1—N1—C5	124.6 (5)	C15—C14—H14	120.4
C1—N1—H1	117.7	C13—C14—H14	120.4
C5—N1—H1	117.7	C14—C15—C16	121.6 (6)
C1—N2—H2A	120.0	C14—C15—H15	119.2
C1—N2—H2B	120.0	C16—C15—H15	119.2
H2A—N2—H2B	120.0	C17—C16—C15	119.0 (6)
C7—N3—C11	124.3 (4)	C17—C16—H16	120.5
C7—N3—H3A	117.9	C15—C16—H16	120.5
C11—N3—H3A	117.9	N5—C17—C16	118.8 (6)
C7—N4—H4A	120.0	N5—C17—C18	115.7 (6)
C7—N4—H4B	120.0	C16—C17—C18	125.4 (6)
H4A—N4—H4B	120.0	C17—C18—H18A	109.5
C13—N5—C17	122.8 (5)	C17—C18—H18B	109.5
C13—N5—H5	118.6	H18A—C18—H18B	109.5
C17—N5—H5	118.6	C17—C18—H18C	109.5
C13—N6—H6D	120.0	H18A—C18—H18C	109.5
C13—N6—H6E	120.0	H18B—C18—H18C	109.5
H6D—N6—H6E	120.0	N8—C19—N7	117.9 (4)
C19—N7—C23	124.2 (4)	N8—C19—C20	124.4 (4)
C19—N7—H7	117.9	N7—C19—C20	117.7 (4)
C23—N7—H7	117.9	C21—C20—C19	119.7 (4)
C19—N8—H8A	120.0	C21—C20—H20	120.1
C19—N8—H8B	120.0	C19—C20—H20	120.1
H8A—N8—H8B	120.0	C20—C21—C22	120.3 (4)
N2—C1—N1	118.7 (5)	C20—C21—H21	119.8
N2—C1—C2	124.3 (6)	C22—C21—H21	119.8
N1—C1—C2	117.0 (6)	C23—C22—C21	119.5 (4)
C3—C2—C1	119.8 (7)	C23—C22—H22	120.3
C3—C2—H2	120.1	C21—C22—H22	120.3
C1—C2—H2	120.1	C22—C23—N7	118.6 (4)
C2—C3—C4	120.8 (7)	C22—C23—C24	125.0 (4)
C2—C3—H3	119.6	N7—C23—C24	116.3 (4)
C4—C3—H3	119.6	C23—C24—H24A	109.5
C5—C4—C3	119.4 (7)	C23—C24—H24B	109.5
C5—C4—H4	120.3	H24A—C24—H24B	109.5
C3—C4—H4	120.3	C23—C24—H24C	109.5
C4—C5—N1	118.3 (7)	H24A—C24—H24C	109.5
C4—C5—C6	124.6 (7)	H24B—C24—H24C	109.5
N1—C5—C6	117.0 (6)		
			
C5—N1—C1—N2	−177.8 (5)	C17—N5—C13—N6	−179.1 (5)
C5—N1—C1—C2	1.9 (8)	C17—N5—C13—C14	0.3 (7)
N2—C1—C2—C3	−179.6 (7)	N6—C13—C14—C15	178.8 (5)
N1—C1—C2—C3	0.7 (10)	N5—C13—C14—C15	−0.5 (8)
C1—C2—C3—C4	−3.6 (13)	C13—C14—C15—C16	0.0 (9)
C2—C3—C4—C5	4.1 (14)	C14—C15—C16—C17	0.7 (10)
C3—C4—C5—N1	−1.6 (12)	C13—N5—C17—C16	0.4 (8)
C3—C4—C5—C6	176.5 (8)	C13—N5—C17—C18	179.4 (5)
C1—N1—C5—C4	−1.4 (10)	C15—C16—C17—N5	−0.9 (9)
C1—N1—C5—C6	−179.6 (6)	C15—C16—C17—C18	−179.8 (6)
C11—N3—C7—N4	179.8 (4)	C23—N7—C19—N8	179.6 (4)
C11—N3—C7—C8	−0.3 (7)	C23—N7—C19—C20	−0.4 (6)
N4—C7—C8—C9	−179.6 (5)	N8—C19—C20—C21	179.5 (4)
N3—C7—C8—C9	0.5 (7)	N7—C19—C20—C21	−0.4 (7)
C7—C8—C9—C10	−0.3 (7)	C19—C20—C21—C22	1.3 (7)
C8—C9—C10—C11	0.0 (7)	C20—C21—C22—C23	−1.4 (7)
C9—C10—C11—N3	0.2 (6)	C21—C22—C23—N7	0.5 (6)
C9—C10—C11—C12	−178.7 (5)	C21—C22—C23—C24	−179.3 (5)
C7—N3—C11—C10	0.0 (7)	C19—N7—C23—C22	0.4 (6)
C7—N3—C11—C12	179.0 (4)	C19—N7—C23—C24	−179.8 (4)

Symmetry codes: (i) −*x*+1, −*y*+1, −*z*+1; (ii) −*x*+1, −*y*+1, −*z*.

### Hydrogen-bond geometry (Å, °)

**Table:**

*D*—H···*A*	*D*—H	H···*A*	*D*···*A*	*D*—H···*A*
O1—H1WB···Cl7	0.83	2.28	3.051 (3)	157
N2—H2B···Cl5	0.86	2.65	3.432 (3)	151
N4—H4B···Cl2	0.86	2.48	3.307 (3)	163
N5—H5···Cl7	0.86	2.21	3.059 (3)	168
N7—H7···Cl4	0.86	2.38	3.204 (3)	161
N8—H8B···Cl1	0.86	2.51	3.343 (3)	164
O1—H1WA···Cl3^iii^	0.83	2.49	3.290 (3)	163
N1—H1···O1^iv^	0.86	1.91	2.774 (3)	177

Symmetry codes: (iii) *x*+1, *y*, *z*; (iv) *x*−1, *y*, *z*.
